# The Recent Conventions

**Published:** 1898-10

**Authors:** 


### Editorial.

### THE RECENT CONVENTIONS.

The conventions called to meet at Omaha on the 26th and 30th of August have performed their allotted work and passed into history. The selection of this period of the year, under the supposition that it would be found more agreeable in temperature, was proved a serious mistake, for the excessive heat of a remarkably hot summer was intensified, and life at Omaha was made almost unendurable.

The Association of Faculties met at the appointed time, with but few colleges unrepresented. The work of this body was, upon the whole, satisfactory, and much of value was accomplished. There seemed to be a determination to have this meeting show an advance in every direction; in fact, the writer never before witnessed a more harmonious effort to bring dental college work to a standard consistent with the demands of the period. There was no opposition to the resolution requiring a course of seven months, this being passed with the proviso that a college preferring a four years' course could continue the present term of six months.

The preliminary standard was advanced to the entrance of the second year of a high school, and the examinations for this are to be conducted hereafter by county superintendents of schools. This, in the opinion of the writer, constitutes an element of weakness. While it is well to have these preliminary examinations made by disinterested parties, there is no certainty that these will be made as desired by the officials named. There appeared to be no other way to meet this difficulty, and if found unsatisfactory in the future can be changed.

There was considerable friction manifested upon the admission of certain colleges through local opposition. It is time that this character of antagonism should be eliminated from the Association of Faculties. The duty of this organization should be to receive all schools properly endorsed without regard to the opposition of competitive colleges. It is idle to expect that dental colleges will cease to multiply under present State laws, and it is, therefore, the part of wisdom to have these, at the earliest possible moment, under the control of this body. It is recognized that the number of dental colleges is far beyond present needs, but it is not the province of the Faculties to limit the number. While the force of this organization is of a purely moral character, it has held a controlling power over its membership far superior to legal restraints, hence factional opposition to admission should be discountenanced.

The report of the "Committee on Recognition of Degrees in Foreign Countries," Dr. W. C. Barrett, chairman, was in many respects an astounding revelation. It will be published, and should then receive the careful reading of the dental profession. It was shown that in Chicago alone there were a number of legalized affairs, not colleges in any sense, making a business of manufacturing diplomas, not only in dentistry, but in all departments of human knowledge. These were kept for sale only in foreign countries, none being sold in the United States. The details were absolutely shocking to those present not previously informed. There could be, however, no dispute as to the correctness of the charges made. The Association took immediate steps to endeavor to stop this nefarious traffic, but this cannot be done without legal evidence. Unfortunately, the Constitution of the State of Illinois years ago made the procurement of a charter a very easy matter. The motive was apparently a good one as applied to general business, but under it unscrupulous men saw their opportunity and, driven from other States, settled here with the result that these fraudulent diplomas have found their way into all parts of the Old World. Dentists in foreign countries will now, it is hoped, aid the committee in the procurement of evidence against these men, for it *must come from the other side*, and cannot be had in the United States. It took years of continued effort to secure legal evidence sufficient to convict the notorious Buchanan, but it ought not to require much time or ingenuity to accomplish more speedily the same result in these cases.

The National Dental Association met with a large attendance from all sections of the country, the Pacific coast being well represented. It would be pleasant to write that these were well repaid for the time and money expended, but the truth must be stated that the meeting was practically a failure. This was the general sentiment, and some even went further and asserted that the National had been strangled in its birth. This, however, is not the opinion of the writer. There were many reasons why this meeting should have been unsatisfactory. The war diverted the minds of men into other channels. It is very certain that the arts of peace have not been cultivated the past year. Then, again, the heat of the past month, almost unexampled in severity, has unfitted every one for work upon matter suitable for such a meeting. In addition to these the heat at Omaha made attendance at the sessions a period of suffering. Making due allowance for these obstacles, the fact remains that there was an evident lack of preparation for this meeting. The papers were few, but several of these were of excellent quality. The one fault of this meeting was a too lengthened attention to routine work. The first day was almost entirely occupied in settling questions that should not have been brought before the main body. The second day was somewhat better in this respect, and the third, and last day, was mainly taken up by selection of officers and place of meeting. From this brief outline it will be quite evident that those who took the long, hot journey received but poor remuneration for the effort.

It was also evident that the members present felt that something must be done to save the organization, and this resulted in a spontaneous effort to inject a more vigorous element into it. This feeling resulted in the election of one of the younger men as President, Dr. Burkhart, of Batavia, N. Y. His well-known ability insures the meeting for next year from disaster. If, however, his energy fails to rescue it, then the dentists of the country must look forward to an entire reorganization of the national body upon a different plan from that heretofore adopted.

When the time comes for another meeting in the West more attention must be given to the place than was accorded it at Old Point Comfort. Omaha would not have been objectionable at any other season of the year, but it has been made evident as very unsuitable during the warm period. The hotel accommodations were not equal to expectations, and in some respects were of a decidedly inferior character. The Executive Committee, in the future, should exercise more care in the examination of hotels, and thus avoid the discomfort and annoyance many were subjected to on their first arrival in that city.

Niagara Falls was selected as the place of meeting in 1899. This was especially desired by the Western members. The best meetings of the "American" were those held at this place, and now that the President resides in the near neighborhood, much of the difficulty attending the recent meeting will be obviated. It is, therefore, safe to predict a gathering next year worthy the dental profession and in accord with its past history.

